# Long Non-Coding RNA ucoo2kmd.1 Regulates CD44-Dependent Cell Growth by Competing for miR-211-3p in Colorectal Cancer

**DOI:** 10.1371/journal.pone.0151287

**Published:** 2016-03-14

**Authors:** Xiaoli Wu, Xixi He, Shi Li, Xiaoqun Xu, Xiangjian Chen, Hua Zhu

**Affiliations:** 1 Department of gastroenterology, The First Affiliated Hospital of Wenzhou Medical University, Wenzhou, Zhejiang, P.R. China; 2 Department of Urology, The First Affiliated Hospital of Wenzhou Medical University, Wenzhou, Zhejiang, P.R. China; 3 Operating room, The First Affiliated Hospital of Wenzhou Medical University, Wenzhou, Zhejiang, P.R. China; 4 Department of endoscopic surgery, The First Affiliated Hospital of Wenzhou Medical University, Wenzhou, Zhejiang, P.R. China; 5 Department of Obstetrics and Gynecology, The First Affiliated Hospital of Wenzhou Medical University, Wenzhou, Zhejiang, P.R. China; Medical College of Soochow University, CHINA

## Abstract

In addition to protein-coding genes, the human genome makes a large amount of noncoding RNAs. Long non-coding RNAs (lncRNAs) have been described as the largest subclass of the non-coding transcriptome in human noncoding RNAs. In recent years, lncRNAs have been considered to be the key regulators of tumor behavior. In this study, based on previous research, we investigated the expression and biological role of a newly identified cancer-related lncRNA, *lncRNA-uc002kmd*.*1*. We analyzed the relationship between *lncRNA-uc002kmd*.*1* and colorectal cancer (CRC) in a total 45 CRC and paired adjacent, non-tumor tissue samples. We found that *lncRNA-uc002kmd*.*1* expression was usually highly expressed in carcinoma compared with the tissue adjacent to the carcinoma. Through a series of experiments, the results showed that *lncRNA-uc002kmd*.*1* regulates CD44 as a molecular decoy for miR211-3p. Our data indicated that the overexpression of *lncRNA-uc002kmd*.*1* enhanced cell proliferation in CRC.

## Introduction

Colorectal cancer (CRC) is an important public health problem globally and remains a major cause of cancer mortality in the developed world, largely due to its propensity to metastasize[1]. It is the second most common cancer diagnosis among women and the third most common among men[2]. Although the achievements in colorectal cancer research are remarkable, new and effective therapeutic strategies are still needed. The search for new biomarkers for metastatic progression in colorectal cancer is urgent.

Long non-coding RNAs (lncRNAs), which comprise non-coding transcripts of more than 200 nucleotides, have been described as the largest subclass of the non-coding transcriptome in humans [3]. A significant amount of past research has focused on the regulatory and structural role of lncRNAs in several important biological processes such as chromosome inactivation, cell differentiation, genomic imprinting and development, and cell proliferation [4, 5]. In recent years, with a wide role for lncRNA in cancer development, increasing numbers of studies on its association with the occurrence of cancer development (e.g., studies regarding CRC[2, 6], esophageal squamous cell carcinoma (ESCC)[7], and gastric cancer (GC)[8]) have been published. Despite these findings, our current knowledge about the expression patterns and functional role of lncRNAs in CRC remains limited.

Recently, one study found a novel lncRNA in GC, *lncRNA-uc002kmd*.*1*, also termed *GAPLINC*. This lncRNA forms a molecular decoy for miR211-3p, which targets CD44 for degradation, and the overexpression of *lncRNA-uc002kmd*.*1* could therefore improve CD44 expression by competing for miR-211-3p, which builds a model for *lncRNA-uc002kmd*.*1*-mediated cell migration and proliferation in gastric cancer[9, 10]. As one of the most putative stem cell markers, CD44 plays a key role in many cellular processes, including cancer cell growth and migration[11]. Additionally, previous studies have shown that CD44 is a well-known stem cell marker for CRC [12].

On the basis of this information, we hypothesized in this study that *lncRNA-uc002kmd*.*1* might play a similar role in CRC. To test this hypothesis, we deduced a way to delineate the transcriptional aberration of the *lncRNA-uc002kmd*.*1* between CRC and paired adjacent, non-neoplastic tissues.

## Materials and Methods

### Study subjects

Homogenous Han Chinese comprised the subjects participating in this study. Forty-five CRC and corresponding adjacent tissue samples were obtained from patients at the First Affiliate Hospital of Wenzhou Medical University (Wenzhou). There were no restrictions on age, stage of CRC, sex or histology. Upon recruitment, each participant was scheduled for an interview using an epidemiological questionnaire, after providing written informed consent. The Medical Ethics Committee of Wenzhou Medical University approved this study. The clinical characteristics of all the patients are listed in Table 1, as described in detail previously[13].

**Table 1:** Baseline demographic and clinical characteristics of study populations.

Characteristics	CRC	
	N	(%)
**Age(years)**		
≤40	4	(8.9)
40–60	16	(35.5)
≥60	25	(55.6)
**Sex**		
Male	22	(48.9)
Female	23	(51.1)
**Tumor invasion**		
T1	5	(11.1)
T2	9	(20)
T3	31	(68.9)
**Family history**		
Yes	6	(13.3)
No	39	(86.7)
**Smoking**		
Never	30	(66.7)
Ever	15	(33.3)
**Drinking**		
Never	21	(46.7)
Ever	24	(53.3)
**Pathological type**		
Highly	23	(51.1)
Moderately	12	(26.7)
Low	10	(22.2)
**Stage**		
I	9	(20)
II	16	(35.6)
III	20	(44.4)

### Cell culture

Human colorectal cancer cell lines, HCT116 and SW480, were purchased from the Cell Bank of Type Culture Collection of the Chinese Academy of Sciences, Shanghai Institute of Cell Biology, and were passaged for less than 6 months. The cell culture procedures have been published elsewhere[13]. Cells were cultured at 37°C in 5% CO_2_ in RPMI-1640 medium supplemented with 10% fetal bovine serum, penicillin and streptomycin in a 10-ml culture dish.

### RNA extraction and real-time quantitative polymerase chain reaction

TRIzol^®^ reagent (Invitrogen) was used to isolate total RNA from the cells and tissues, according to the manufacturer’s instructions. The relative gene expression of *GAPLINC* was determined using the ABI Prism 7500 sequence detection system (Applied Biosystems, Foster City, CA, USA). *GAPDH* was used as an internal standard control, and all the reactions were performed in triplicate [14, 15]. The primers used for qPCR amplification included the forward GTTTCCTGGAAGGGCATTTT and the reverse CAATCAGGGCTCTTGGACTC.

### Construction of reporter plasmids

The method for the construction of reporter plasmids has been published elsewhere [9]. The pGL3 promoter vector (GENECHEM) was used to construct the plasmids pGL3- *lncRNA-uc002kmd*.*1*-3’UTR (the plasmid containing *lncRNA-uc002kmd*.*1* 3’UTR) and pGL3- CD44-3’UTR. All the constructs were verified by DNA sequencing.

### Transient transfections and luciferase assays

HCT116 and SW480 were transfected with the reporter plasmids using Lipofectamine 2000 (Invitrogen, CA, USA), according to the manufacturer’s instructions. Use of the Dual-Luciferase Reporter assay system (Promega, Madison, WI, USA) to measure the luciferase activity has been published elsewhere[16]. Three independent experiments were conducted and each group included 6 replicates.

### Actinomycin D assay

HCT116 and SW480 were transiently transfected using Lipofectamine 2000 (Invitrogen) and co-transfected with miR-211-3p, as indicated, for 24 h; the cells were then exposed to actinomycin D (Sigma, St Louis, MO). The cells were harvested and the stability of the *lncRNA- uc002kmd*.*1* mRNA was analyzed using quantitative reverse transcriptase PCR (qRT-PCR), as previously described[13].

### Western blot

Western blot analysis was conducted to assess CD44 and â-action expression, as previously described[13]. The Western blotting analysis was repeated at least three times.

### Cell viability assay

The Cell Counting Kit-8 (CCK-8) system (Dojindo Laboratory, Kumamoto, Japan) was used to measure cell viability, according to the manufacturer’s instructions[16]. There were 6 replicates for each group, and the experiments were repeated at least 3 times.

### Statistical analyses

The correlation between the expression of *lncRNA-uc002kmd*.*1* and the *CD44* gene in CRC tissue was assessed using one-way analysis of variance and linear regression models. Differences between the groups were assessed using paired, 2-tailed Student’s t-test. A *P*-value of <0.05 was considered statistically significant.

## Results

### Cellular characterization of *LncRNA-uc002kmd*.*1*

**Fig 1:**
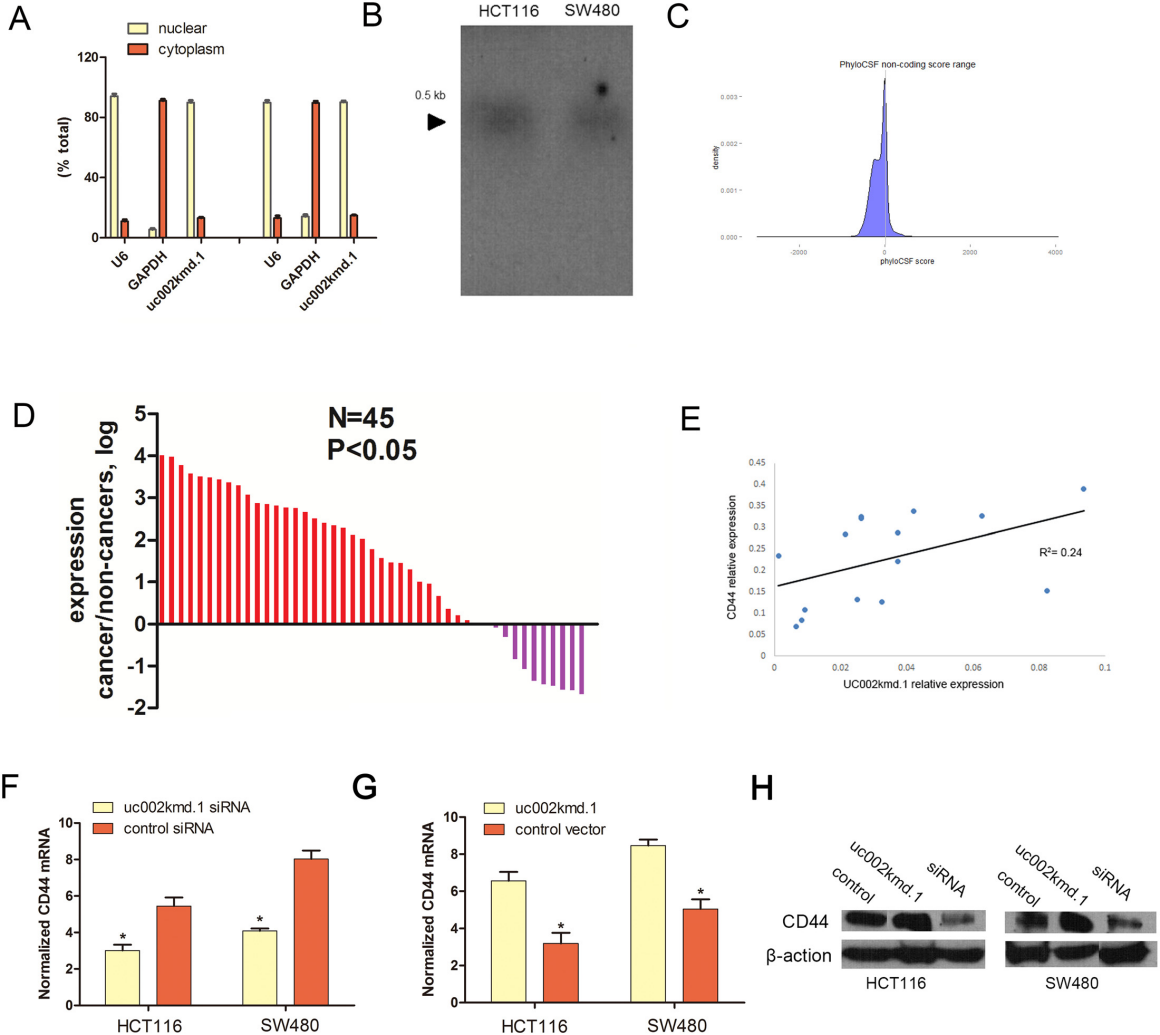
Cellular and molecular characterization of *lincRNA-uc002kmd*.*1*. (A) The levels of nuclear control transcript (U6), cytoplasmic control transcript (GAPDH mRNA) and *lincRNA-uc002kmd*.*1* were assessed by qRT-PCR in nuclear and cytoplasmic fractions. Data are mean ± SEM. (B) Northern blot analysis of *lincRNA-uc002kmd*.*1*expression in CRC cells. (C) Maximum CSF scores of *lincRNA-uc002kmd*.*1* by analysis with PhyloCSF. The score is -178.6075. (D) The *lincRNA-uc002kmd*.*1* was expressed at a higher level in CRC tissues compared to match CRC adjacent tissues. The expression level of *lincRNA-uc002kmd*.*1* was analyzed by qRT-PCR normalized to *GAPDH*. Data are represented as mean±SEM from three independent experiments. (E) The linear correlations between the *lincRNA-uc002kmd*.*1* expression levels and CD44 mRNA were tested. The relative expression value was normalized by *GAPDH* expression level. (F, G) *lincRNA-uc002kmd*.*1*expression significantly affected CD44 mRNA expression. Knockdown of *lincRNA-uc002kmd*.*1* decreased CD44 expression, while ectopic expression of GAPLINC increased CD44 mRNA level. (H) The protein levels of CD44 was assessed in CRC cells (HCT116 cells and SW480 cells) by Western blot.

To determine the cellular localization of *lincRNA-uc002kmd*.*1*, the levels of the nuclear control transcript (*U6*) and cytoplasmic control transcript (*GAPDH* mRNA) were detected by RT-qPCR in the nuclear and cytoplasmic fractions, respectively. For the HCT116 cell line, qRT-PCR analysis revealed that (mean ± SEM) 89.8% *lincRNA-uc002kmd*.*1* was detected in the nuclear fraction, and 13.1% was situated in the cytoplasmic fraction. Similar results were obtained with the SW480 cell line, specifically, 89.2% and 11.8% *lincRNA-uc002kmd*.*1* was detected in the nuclear fraction and the cytoplasmic fractions, respectively (Fig 1A).

*LincRNA-uc002kmd*.*1* could be detected as one transcript of the expected size by northern blotting (Fig 1B) in the CRC cells. At the same time, we examined the coding potential of *lincRNA-uc002kmd*.*1* using PhyloCSF, the PhyloSCF score is -178.6075, this result showed the low coding potential of *lincRNA-uc002kmd*.*1*(Fig 1C).

### *LncRNA-uc002kmd*.*1* is up-regulated in CRC tissues

The expression level of *lncRNA-uc002kmd*.*1* was examined using real-time PCR in 45 pairs of CRC tissue and matched adjacent normal tissue. Detailed clinical features are presented in Table 1. The *lncRNA-uc002kmd*.*1* transcripts were expressed at higher levels in the tumor tissue compared with adjacent normal tissue (Fig 1D).

### Association of *lncRNA-uc002kmd*.*1* and *CD44* in CRC

We tested the correlation between *lncRNA-uc002kmd*.*1* and *CD44* in another 15 pairs of CRC adjuvant non-cancerous tissues. The results showed that patients with higher *lncRNA-uc002kmd*.*1* expression levels in CRC tissue displayed substantial up-regulation of CD44 (*R*^*2*^ = 0.24, *P* < 0.05; Fig 1E).

Using specific siRNAs, knockdown of *lncRNA-uc002kmd*.*1* substantially decreased CD44 expression, whereas overexpression of *lncRNA-uc002kmd*.*1* enhanced CD44 mRNA levels (Fig 1F and 1G). Western Blot results consistently showed that knockdown of *lncRNA-uc002kmd*.*1* decreased CD44 protein levels in the HCT116 cell line, while overexpression of lncRNA-uc002kmd.1 increased CD44 protein levels. Similar results were found in the SW480 cell line (Fig 1H).

### *LncRNA-uc002kmd*.*1* regulates *CD44* expression by competing for miR-211-3p

**Fig 2:**
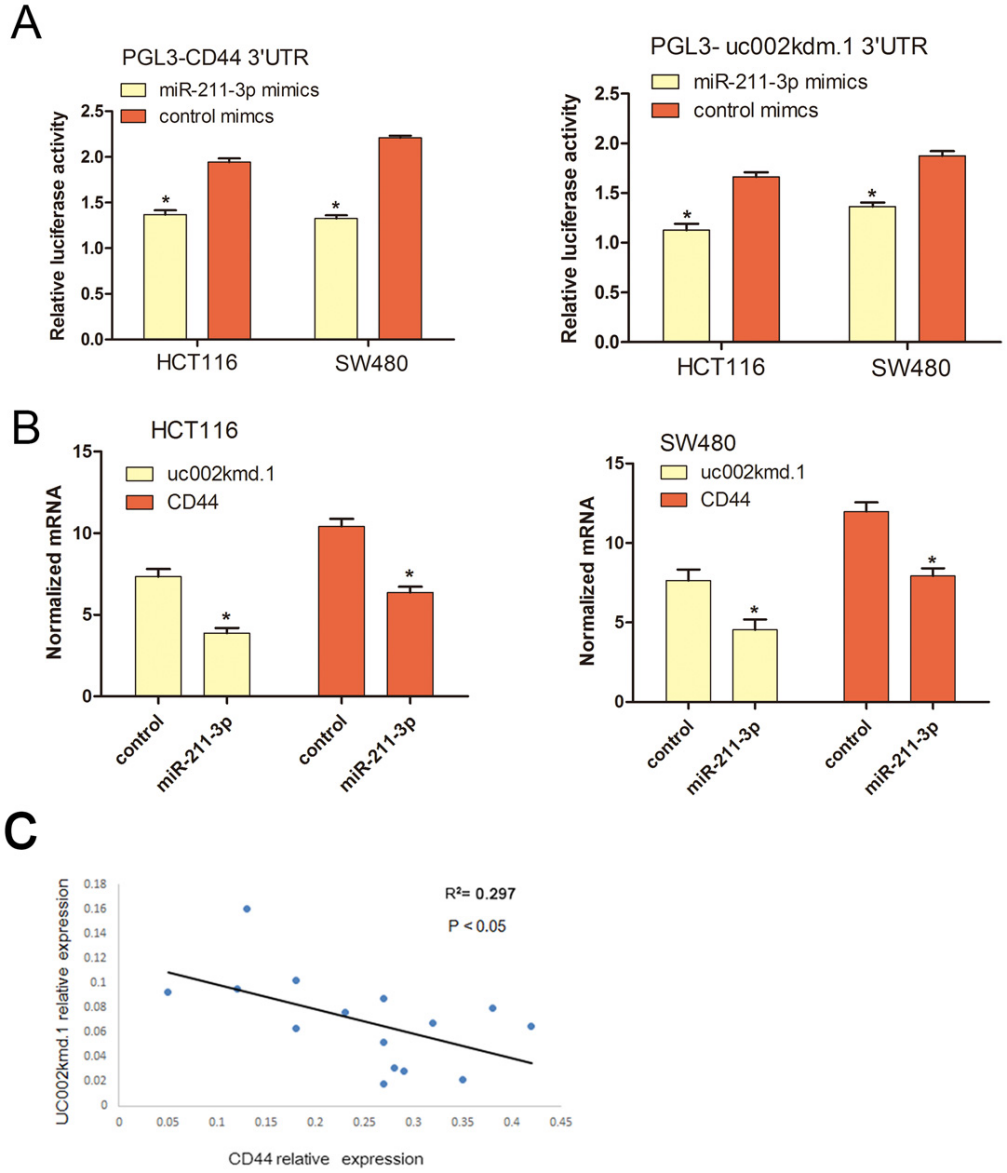
The relationship between miR-211-3p, *lincRNA-uc002kmd*.*1* and *CD44*. (A)Both *lincRNA-uc002kmd*.*1* and CD44 are targeted by miR-211-3p. MiR-211-3p significantly decreased the luciferase signals of both *lincRNA-uc002kmd*.*1* and CD44. (B) The CD44 and *lncRNA-uc002kmd*.*1* levels were significantly decreased. Data are mean±SEM, normalized to *GAPDH*. (C) The negative correlations between the *lincRNA-uc002kmd*.*1* expression levels and CD44 mRNA were tested.

Coincidentally, the miRNA known as miR-211-3p was the predicted target for both *lncRNA-uc002kmd*.*1* and CD44. To verify the role of miR-211-3p in the CRC cells, we cloned the 3’ UTR of CD44 and *lncRNA-uc002kmd*.*1* downstream of a luciferase gene and co-transfected these reporters with miR-211-3p mimics in the CRC cells. As shown in Fig 2A, miR-211-3p significantly decreased the luciferase signals of both reporters. Moreover, we measured the CD44 and *lncRNA-uc002kmd*.*1* levels in the CRC cells after treating with the miR-211-3p mimics. As expected, the CD44 and *lncRNA-uc002kmd*.*1* levels were significantly decreased (Fig 2B). Besides this, we test the expression level of CD44 and miR-211-3p in 15 pairs CRC tissues, the results demonstrate a negative correlation between miR211-3p and CD44 expression levels (*R*^*2*^ = 0.24, *P* < 0.05; Fig 2C).

**Fig 3:**
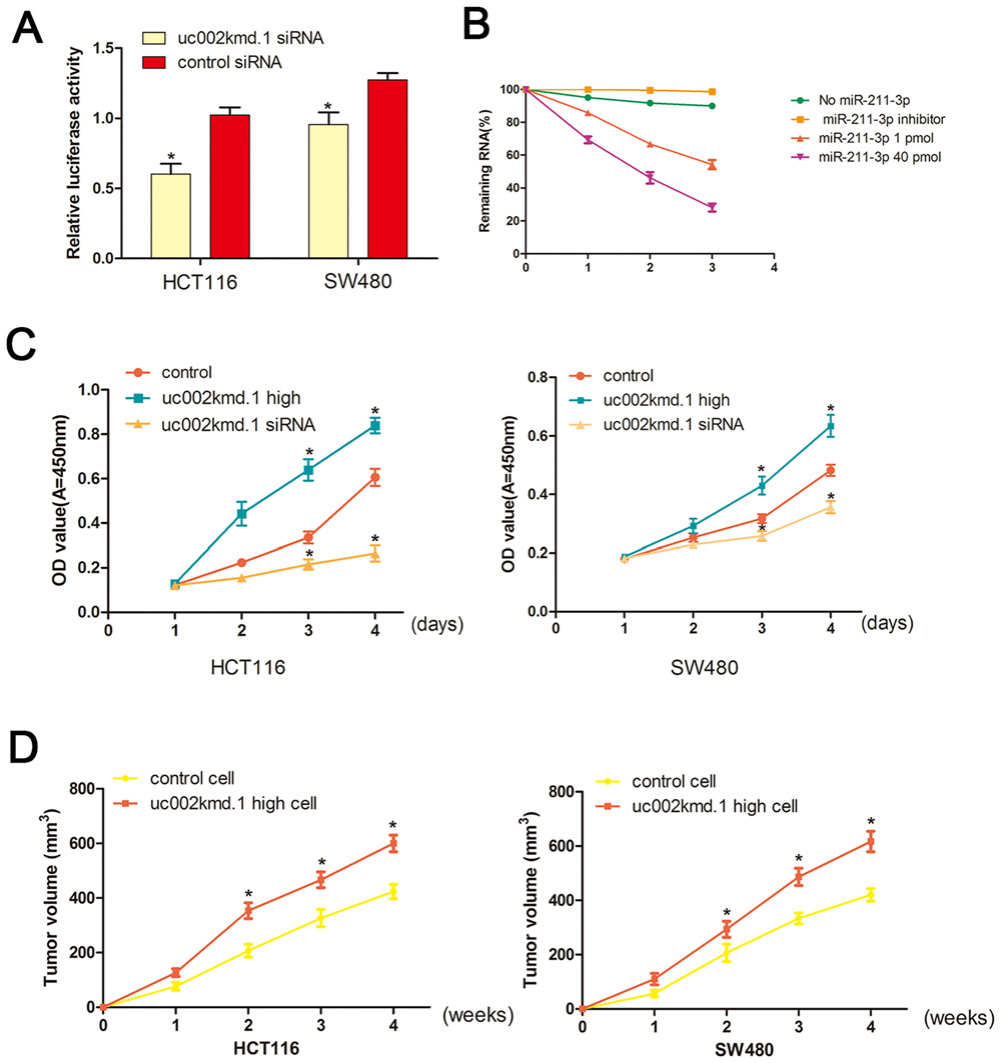
*lincRNA-uc002kmd*.*1* mediated cell proliferation in CRC cells. (A) The reporter vector was co-transfected to CRC cells, which were treated by *lncRNA-uc002kmd*.*1* siRNA or control siRNA. The luciferase signal was significantly decreased. (B) Cells were harvested and the stability of *lncRNA-uc002kmd*.*1* mRNA was analyzed by qRT-PCR relative to time 0 after blocking new RNA synthesis with actinomycin D; data are mean±SEM, normalized to *GAPDH*. (C) HCT116 and SW480 cells were seeded in 96-well plates after been transfected, and cell proliferation was performed daily for 3 days using the CCK-8 assay. Six replicates for each group and the experiment repeated three times. Data aremean±SEM. *P<0.05 compared with controls. (D) The data showed tumor volumes of xenografts in each group 4 weeks after subcutaneously implanted stable CRC cells. Mean tumor volumes from six nude mice of each group are shown at different time points. *P<0.05 compared with controls.

After knockdown of *lncRNA-uc002kmd*.*1* by siRNA, we cloned the 3’UTR region of CD44 into a luciferase reporter and co-transfected the construct with *lncRNA-uc002kmd*.*1* siRNA or control siRNA. The results then showed that knockdown of *lncRNA-uc002kmd*.*1* significantly reduced the intensity of the luciferase. All these results suggested that miR-211-3p could target *lncRNA-uc002kmd*.*1* and CD44 and that *lncRNA-uc002kmd*.*1* is required for the abundant expression of CD44 (Fig 3A).

### *LncRNA-uc002kmd*.*1* modulates cell growth

We further investigated the effect of *lncRNA-uc002kmd*.*1* on cell proliferation in *vitro*. CRC cells were transfected with miR-211-3p mimics or with an miR-211-3p inhibitor, and the transcript levels of the *lncRNA-uc002kmd were* down-regulated after RNA synthesis was blocked with actinomycin D in the presence of miR-211-3p (down-regulated from 100% to 54% ± 3.2% in the presence of 1 pmol miR-211-3p; and down-regulated from 100% to 28%±3.2% in the presence of 40 pmol miR-211-3p).

We performed CCK-8 assays to test the effects of *LincRNA-uc002kmd*.*1* on cell proliferation in CRC cells. We observed a consistent increase in cell proliferation of the HCT116 (38% increase) and SW480 (31% increase) cell lines when *LncRNA-uc002kmd*.*1* was overexpressed at physiological levels through CCK-8 assays, when compared to the control. Whereas *lncRNA-uc002kmd*.*1* down-regulated the cells, the proliferation of the HCT116 (50% decrease) and SW480 (27% decrease) cell lines was consistently decreased (Fig 3C).

### *LncRNA-uc002kmd*.*1* accelerate tumor growth in xenograft

To examine the biological significance of *LncRNA-uc002kmd*.*1* on tumor growth, xenograft were subcutaneously injected with CRC cells. As is shown in Fig 3D, the growth of tumors from up-regulated *LncRNA-uc002kmd*.*1* xenografts was significantly increased compared with that of the control xenografts: 423.3 ± 71.6 mm^3^ versus 600 ± 17.6 mm^3^ for HCT116 cells (*P* < 0.05); and 420 ± 70.8 mm^3^ versus 616.7 ± 21.7 mm^3^ for SW480 cells (*P* < 0.05), respectively.

## Discussion

In this study, we identified the *lncRNA-uc002kmd*.*1* in CRC and found that it was dramatically up-regulated in CRC tissue using the reverse transcriptase polymerase chain reaction assay, indicating the potential function of *lncRNA-uc002kmd*.*1* in CRC. A series of experiments have illustrated the correlation between *lncRNA-uc002kmd*.*1*, miR-211-3p and CD44, concluding that *lncRNA-uc002kmd*.*1* regulates CD44-dependent cell growth by competing for miR-211-3p in colorectal cancer. Our findings indicate the important role of *lncRNA-uc002kmd*.*1* during CRC tumorigenesis.

LncRNAs are emerging as key players in various fundamental biological processes and a growing amount of research has proposed that lncRNAs are important players in cancer development [17–19]. The most well-known lncRNA, HOTAIR, is up-regulated in gallbladder cancer (GBC) that leads to tumor metastases through altered methylation of histone H3 lysine 27 (H3K27) and gene expression [20, 21]. *Linc-POU3F3* is increased in ESCC samples, which, through interactions with *EZH2* to promote methylation of *POU3F3*, then promote tumor development [22]. Other than these annotated-lncRNA, non-annotated lncRNA has been having the desired effect on the development of many malignant tumors, such as colorectal cancer-associated lncRNA (CCAL) promoting CRC progression by the activated Wnt/β-catenin pathway [23]. A recent report found that *lncRNA-uc002kmd*.*1*, also known as GAPLINC, was up-regulated in GC and also displayed considerable predictive effects in the diagnosis and prognosis of gastric cancer, whereas, in our study, *lncRNA-uc002kmd*.*1* was also involved in the promotion of CRC development.

Next, we investigated the mechanisms by which *lncRNA-uc002kmd*.*1* exerts its function in malignant CRC phenotypes. Our results clearly showed that when silencing *lncRNA-uc002kmd*.*1* expression, CRC cell proliferation was inhibited. Our data also affirmed that *lncRNA-uc002kmd*.*1* forms a molecular decoy for miR211-3p. It is common knowledge that miRNAs are short RNA sequences, though target sequences of the 3'UTRs of mRNAs then negatively manipulate gene expression. MiRNAs are involved in various biological processes, such as cell proliferation, development, differentiation and metabolism. In general, miRNAs play a pivotal role in gene regulation, mainly by targeting abundant protein-coding genes. Increasingly, however, more research has found that miRNAs can also perform their function by targeting lncRNAs [24]. The theory of competitive endogenous RNA showed that coding genes and lncRNAs can regulate each other through their competition for miRNA binding [25]. According to this theory, lncRNAs may exert their influence on targets by serving as decoys for miRNAs.

In this paper, we identified the CD44 protein as an important part of the *lncRNA-uc002kmd*.*1*- miRNA regulatory network. Using the luciferase reporter assay, we found that CD44 is repressed by miR-211-3p, and this function is attenuated by *lncRNA-uc002kmd*.*1* overexpression. We also found that the level of CD44 protein gradually increased with increasing levels of *lncRNA-uc002kmd*.*1*.

CD44 is a cell surface transmembrane glycoprotein that plays a significant role in a number of biological functions [26]. Previous studies have demonstrated that CD44 plays a critical role in AML development and the variations of CD44 could affect its expression, leading to varying risks of AML [15]. Additionally, CD44 is an important stem cell marker for multiple solid tumors, which leads to the development and progression of cancer [27]. CD44 could be used as a novel marker for the characteristics and management of many tumors such as GC [28] or CRC [11]. Recently, many studies have demonstrated a significant correlation between the level of *CD44* expression and breast cancer tumorigenicity, which highlights the important role of *CD44* in tumor progression and metastasis [11, 29, 30]. In our study, we showed that miR211-3p binds to the 3’UTR region of the CD44, targeting CD44 for degradation. Through the miR211-3p decoy, the *lincRNA-uc002kmd*.*1* increases CD44 expression.

Collectively, our findings indicate that *lincRNA-uc002kmd*.*1* may improve CD44 expression by competing for miR-211-3p, subsequently mediating cell migration and proliferation in CRC.
